# New Appliances and Things Medical

**Published:** 1893-06-24

**Authors:** 


### NEW APPLIANCES AND THINGS MEDICAL.

[All preparations, appliances, novelties, etc., of which a notice is desired, should be sent for the Editor, to care of The Manager, 428, Strand, London, W.C.]

### AMINOL.

(THOMAS HODGKINSON, PRESTONS AND KING, 81, BISHOPSGATE STREET WITHOUT, E.C.)

This is a solution of the gas produced by the action of caustic lime on the brine products resulting from the waste materials of certain food manufactures. It is essentially the adaptation of the amines method of sewage disinfection to domestic and medical purposes. Two solutions are sold, one for general purposes such as washing down floors and drain cleansing, and the other for surgical and internal uses. Accompanying the samples forwarded to us is a report by Dr. Klein on the effect of the disinfectant on germ destruction. One deals with the ordinary germs of disease of varying kinds, and the other with the effect of aminol on the cholera bacillus. Both reports are highly interesting. In the latter, Dr. Klein states that aminol is totally destructive of the organism. In the former he shows that the solution is an extremely powerful bactericide. We have experimented on the antifermentitive and also on antiputrefactive actions of the two solutions, and find that in both the aminol is a powerful agent in the prevention of the changes indicated Like most disinfectants it has a strong odour, but unlike a large number it is not poisonous. Solution B is especially designed for internal administration as well as for external application. Dr. Campbell Pope speaks highly of its action in diphtheria, and there is a considerable body of clinical evidence showing its useful application as a germ-destroying agent in medicine. We welcome the appearance of a new disinfectant, as there are always cases in which one kind is unsuitable and for which we must find an alternative application.

### ELIXIR PICIS CO. (LAYNE.)

The Layne Medical Manufacturing Company, 6, Benson's Chambers, Ludgate Hill, have forwarded to us a specimen of the above. It contains in each drachm the soluble parts of one grain of purified wood tar. This is dissolved in a menstruum which is not only well flavoured, but which assists the tar in its action. The whole forms a very palatable cough linctus which will be useful in those conditions for which tar is indicated, more especially in the bronchial and flatulent cases which are so troublesome both to patient and doctor.

### OFFENSIVE ODOURS.

To free the hands from the smell of iodoform, creosote, or guiacol, wash them with water in which linseed meal has been boiled and drained off. Objects smelling of iodoform should be washed in tar water to which has been added some essence of winter green. Rooms smelling of creosote or iodoform can be deodorized by burning coffee berries in them. Pills of creosote over which freshly ground coffee has been sprinkled lose their disagreeable odour.

